# A School of Mechanical Dentistry as a Means for the Cultivation of the Mind

**Published:** 1904-09

**Authors:** C. M. Wright

**Affiliations:** Cincinnati.


### A SCHOOL OF MECHANICAL DENTISTRY AS A MEANS FOR THE CULTIVATION OF THE MIND.

By C. M. Wright, D. D. S., Cincinnati.

(Written for *The American Journal of Dental Science.*)

President Eliot is reported by the daily papers to have said in an address delivered in Cincinnati: "Give to each of a thousand boys a block of wood and a penknife and tell them what to make of the block; from the results I will guage the Moral character of each boy."

Many thinking men engaged seriously with the complex problems of Education have arrived at the conclusion that the hand and the eye afford the best route for normal stimulation of the fundamental properties of the mind.

Attention, perception, reflection, memory, judgment and will can be more readily and successfully exercised by properly selected manual studies than by any other known method. This is gradually becoming a leading idea in all departments of education.

The Harvard or Case system method of teaching Law, is a modification of it. It is at variance with the ancient doctrine held by philosophers and teachers and still handicapping us by the force of habit and tradition.

Mathematics language and the sciences have been taught for centuries, not for the practical value of geometry, Greek and astronomy but for their value in the cultivation of certain qualities of the mind. The early philosophers scorned the idea of use in connection with pure mathematics.

It is believed by modern educators presidents of colleges, teachers, and psychologists that culture can be attained by practical studies and that the training of the mind can be more directly and logically effected by manual and technical than by the older theoretic systems. The practical idea has been slowly but steadily gaining in form and substance since the days of Francis Bacon. The answer to the question of how to make every pupil a student and every student a lawyer, or a doctor, or a wise man, by any system has not been found.

The teacher is a more important factor than a curriculum. This is lost sight of in our desire to arrange plans and methods and grades that may be universal in application.

It is the tendency of the day to lay out cut and dried patterns for schools, as we do tables of diets for infirmaries, hospitals and camps.

The ideal teacher of all ages, past and present, with or without a system, and whether he be old or young, is a leader to draw out the faculties of the student. He holds and develops attention, awakens perception and reflection and stimulates the student to exercise his own judgment. He does this whether his branch is mathematics, language or mechanical dentistry, even though somewhat hampered by transient pedagogic notions. The dental college is subject to all the advantages and disadvantages of the tendency of the times. Marked changes have occured in methods since the old Baltimore and the Ohio College of Dental Surgeons opened their doors. The dental college of today is a part of a general system of state and university education. A boy is expected to have a graded course of mental gymnastics from his sixth year, through public and high school or in some equivalent before he begins his professional and special education, and the dental college with its modern text books, its entrance, midterm and final examinations, its roll call and monitor system, its rules and bylaws, is today very much like a city public school. It belongs to the system.

The old college smilingly opened wide its doors to all who were athirst for knowledge. The practicing dentist, the "student" from some private office, the silversmith, the house carpenter, the preacher, and whoever might decide that he wanted to fit himself for the practice of dentistry could find welcome within the sacred walls. The high school graduate came with this mixed class of applicants, as did occasionly the college graduate with his baccalaureate appendage. The text books were treatises and essays. The teachers were lecturers and demonstrators enthusiastic in their desire to impart instruction and advance the cause of dental education. They willingly left their offices and gave time, energy and money to the work of teaching. The result of this sacrifice and devotion on the part of these early teachers is the dentistry of today. The heterogeneous class of dental students left the colleges as educated men and have borne the banner of the profession proudly before the world. They have been, too, our most distinguished scholars and writers and teachers in the part.

Does the high school and the college prepare a boy for the study of dentistry? Will the "A. B." applicant outrank the old jeweler, or house carpenter after his completed college course?

I shall not attempt to answer these questions, but after many years of observation as a teacher of all sorts and conditions of dental students I am not so sure about the value of the kind of qualifications aimed at by some of our leading educators. This state of mental uncertainty has forced me to watch repeatedly and eagerly for examples of the development of students intellectually and morally, during the college course, no matter what previous schooling or lack of it, antedated their admittance. Like an organic ferment, after the infection, and the period of incubation, the resulting fermentation in my mind is the notion, that the dental schools should do some of their own preliminary training and begin earlier with those who intend practicing dentistry. To accomplish this we must establish a preparatory course of study under the direction of able teachers —a sub-freshman course, lasting one or two years. In this boys of fifteen, sixteen or seventeen years of age might enter and begin technical study in the laboratories. Mechanical dentistry should be the course. The hands and eyes and mind should be trained at the bench, at the forge, at the lathe and at the furnace in the many and various departments offered by this broad study. Practical chemistry, practical mettallurgy, practical ceramics and all that these embrace should be taught. I need not specify the breadth and width of the fields included in mechanical dentistry. We would train the boy technically and mentally—the morals would follow. The man with an educated judgment will be a moral man. At the proper age and with satisfactory evidence of mental and moral training which this preparatory dental school will afford, admit the boy to the college. How about his mathematics and language and general culture? I answer by asking: How has the dentist of today who never had a liberal art college education fitted himself to become the peer of his college bred confrere? By self culture. Open the dentist preparatory training school, then and accept any nice boy who can read and write and educate him in the laboratory. This will raise the standard of dental education as no other course can provided we have accomplished teachers. Afterwards, in the college proper, with the Greek letter fraternities, the debating societies, the glee and athletic clubs, the lectures and discussions a real modern college education can be attained with the attending atmosphere of learning, which is so permeating that it alone has been of incalculable benefit to many a college bred man.

Offer this as a picture of a good method of fitting boys for entrance to the dental college and also as a plan for a school for the training of the mind even if the student does not continue the study or practice of dentistry. I can recite examples of men and women who left the dental college for some other walk in life—some specialty in medicine, or some entirely foreign occupation—who have never regretted the *education* they received in the laboratory and lecture rooms of a dental college. We want students with minds trained before they enter a professional college and I believe that a one, two or three years' course in a dental preparatory school in mechanical dentistry will accomplish this as well as many high school and college courses, and better than the ordinary school work which we accept as sufficient for admittance to the college. My plan is at least in the line of modern scientific methods of education, that is, direct and physiologically economic, instead of diffusive and flavored with dilitantism.

---

After Pulp Removal.—That annoying particle of sensitive tissue at the apex of the root can ordinarily be removed by pumping carbolic acid and chloroform into the root with a smooth broach.—*Chas. E. Parkhurst, International Dental Journal.*

